# Implementation of a Surgical Liaison Service for Elderly Patients: A Single Unit Experience

**DOI:** 10.3390/geriatrics4030046

**Published:** 2019-07-28

**Authors:** Jessica Bennett, Dave Fung, Rachel Hodson, Anil Agarwal

**Affiliations:** University Hospital of North Tees, North Tees and Hartlepool NHS Foundation Trust, Stockton Upon Tees, Durham TS19 8PE, UK

**Keywords:** surgery, geriatrics, NELA, peri-operative care, frailty

## Abstract

Older people over the age of 65 years are recognized as higher risk surgical candidates and it is therefore recommended that their care should be coordinated through a multidisciplinary team (MDT) approach involving senior geriatricians, anaesthetists and surgeons. As one of only a handful of hospitals to implement a liaison service for elderly surgical patients we have seen both quantitative and qualitative improvements in the care delivered. Both co-ordination and continuity of care has improved and overall staff feel that the service forms an integral part of caring for the older surgical patient. Currently only 1% of UK hospitals are meeting targets for implementation of liaison services for their elderly surgical patients. Our surgical liaison service offers consultant led care for older people and is valued amongst users. We would like to share our experiences in the service setup, provision and its subsequent impact on patient care.

## 1. Introduction

The UK health system is currently faced with a rapidly expanding elderly population, with a rise in the number of elderly patients undergoing surgical procedures in both elective and emergency settings [1]. It is well established that elderly people over the age of 65 years are higher risk surgical candidates, owing to their medical comorbidities, and decline in their physiological reserve, nutrition and cognition [2].

Current recommendations support the involvement of a medicine for the care of older people (MCOP) physician in the coordination of peri-operative care for elderly surgical patients, as a multidisciplinary team (MDT) approach has been associated with improved outcomes [3,4]. Despite this, surgical liaison services are not currently delivered in the same proactive and protocol driven manner as orthogeriatric services across the UK. Existing studies have shown that there is desire for closer peri-operative collaboration between surgeons and geriatricians however the current provision of care is often inadequate [5].

Our institution is privileged to be one of the few centres across the UK with a MCOP physician dedicated to providing this service for surgical patients over the age of 65 years. We would like to share our experiences in the set up and provision of a surgical liaison service, and examine its impact on patient care.

The University Hospital of North Tees delivers emergency surgical care for a community population of 400,000, with approximately 60 of its 500-inpatient beds allocated for surgical and urological admissions. There is an average of 5600 surgical admissions per year, of which approximately 25% are over the age of 65 years old. 

Our service was introduced in January 2014 following the appointment of a full time consultant geriatrician with a special interest in surgical liaison. With the help of a specialty registrar they deliver three PA sessions of liaison work per week, totaling around 12 h. They see an average of three new referrals and five follow ups per session. The only costs incurred to the surgical department are related to the three contracted consultant sessions a week, but the physicians are readily accessible outside these hours.

Emergency admissions of elderly surgical patients requiring medical input are highlighted to the MOCP physician in the daily surgical morning handover. Preoperatively, they optimize patients’ medical comorbidities and identify any potential social issues to be addressed in discharge planning. The liaison team then routinely review these elderly patients postoperatively and facilitate timely discharge and rehabilitation. In those who are critically unwell, the MOCP physicians are involved in discussions with family and patient regarding appropriate escalation of care. Referrals to the service are accepted from all healthcare professionals and the process is coordinated effectively via the ward sister, who attends the surgical ward rounds.

## 2. Methods

This retrospective study aimed to examine the effectiveness of our surgical liaison service by comparing patient outcomes before and after the implementation of the service in January 2014. Study participants were identified using theatre records and 57 consecutive patients between the period of June 2013 and December 2014 were included. All of the patients were over the age of 65 and underwent emergency intra-abdominal surgery performed by a general surgeon. Our primary outcome measure was the number of times each patient was reviewed during their inpatient stay by a MCOP physician. Although the dedicated surgical liaison service only became fully funded in January 2014 there were still MCOP physicians working within the trust prior to this date that would have been accessible if requested. Our secondary outcome measures included mortality, length of stay and delay to discharge as well as complication rate and provision of care in the Intensive Care Unit (ITU) We also assessed the rate of allied health professional input in the form of occupational therapy and physiotherapy inpatient assessments. Data was collected and verified by two of the authors (JB and DF) before undergoing statistical analysis. When calculating averages, the median was used, owing to the small size of the dataset. The unpaired *t*-test was used to calculate statistical significance in most cases unless the data was dichotomous in which case Fisher’s exact test was used. Operative severity was categorised into either Major+, Major or other according to the National Emergency Laparotomy Audit guidelines [6]. As the study was deemed to be part of service evaluation individual patient consent was not required. It was registered with the trust audit department and carried out in accordance with local Caldicot guidelines.

In addition to this quantitative data the surgical liaison service was also evaluated qualitatively with questionnaires sent out to 35 health care professionals who have had direct engagement with the service. There were 31 respondents and their responses to three questions have been collated below.

## 3. Results

There were 25 patients in the pre-MCOP cohort versus 32 in the post-MCOP cohort. Although the cohorts were comparable in their ratios of male:females, they differed significantly in age and frailty (Table 1). The post-MCOP group were significantly older (82 vs. 69) and had a greater number of co-morbidities as classified by the American Society of Anaesthesiologists (ASA) physical status classification system (3 vs. 2). A greater percentage of the post-MCOP cohort underwent either Major+ or Major operations when compared to the pre-MCOP cohort (75% vs. 56%), however this difference was not statistically significant.

When evaluating the primary outcome it is clear that the service has led to significantly more surgical inpatients receiving a review by a MCOP physician. Seventy-one reviews were carried out on 32 patients following the introduction of the service versus none in the pre-MCOP cohort.

The introduction of the service also significantly increased the proportion of patients who received input from allied health professionals prior to discharge. In the post-MCOP cohort, 31 out of 32 patients were reviewed by a physiotherapist or occupational therapist prior to discharge versus 11 of the 25 pre-MCOP cohort. This result is statistically significant.

The only other secondary outcome to show a significant difference was the length of stay. This was an average of 11 days in the pre-MCOP cohort versus 16 days in the post-MCOP cohort. In all other secondary outcomes; mortality, delay to discharge, ITU input and complications, there was no statistically significant difference between the two cohorts (Table 2).

Qualitative service evaluation was also undertaken in the form of a staff survey (Table 3). Thirty-one feedback questionnaires were completed which represented an 88% response rate. Ninety seven percent of health care professionals (*n* = 30) felt that elderly patients have more complex care needs and that MCOP reviews were necessary prior to surgery. Ninety percent of those surveyed (*n* = 28) also believed that there was improved access to medical reviews for surgical patients. 

## 4. Discussion

With the implementation of the surgical liaison service, there has been a greater continuity of care for elderly patients, with frequent and regular reviews by the same medical consultant and allied healthcare professionals (Table 2). The results have shown that simply having a MCOP physician within the hospital is not enough, without the service there were no planned reviews of elderly surgical patients. We have shown that with the implementation of a designated service each elderly surgical patient will receive an average of two inpatient visits by an MCOP physician. They also receive increased input from allied health professionals. This may in part explain why the post-MCOP cohort had a longer average length of stay (LOS). Traditionally, there has been an emphasis to discharge patients as soon as they become “medically fit”. We hypothesise that the increase in LOS seen in the post-MCOP could reflect an increase in holistic care and MDT planning by the MCOP physicians and allied health professionals. Patients may have had a prolonged stay due to increased attention to social needs as well as physical rehabilitation. Discharge institutionalization is a recognised risk in the elderly patient with 1 in 3 geriatric patients requiring discharge to an institutional care facility after major surgery [7]. This is often requires complex MDT planning. A MCOP physician is ideally placed to help detect patients that would benefit from additional care on discharge as well as helping to streamline its initiation. 

Although it has been difficult to quantify the benefit of the MCOP liaison service in this small data set many of the qualitative responses received were extremely encouraging. In the free text sections of our questionnaire, respondents commented that overall they felt that the service “dealt with patients proactively rather than reactively” and provided “better access to rehab facilities”. It was also felt that the regular presence of the MCOP physician increased support for ward based junior doctors and aided with discharge planning. Respondents also commented that they felt escalation and resuscitation planning was better addressed since the introduction of the service. 

Although there is no reduction in the incidences of medical complications in the post-MCOP cohort, the presence of a dedicated consultant led liaison service should only indicate better quality care for our patients. In the absence of a comprehensive geriatric assessment (CGA) and time for medical optimization in the emergency setting, these quantitative outcome measures can misrepresent the effectiveness of a surgical liaison service [8].

We acknowledge that a proactive CGA service has led to improved outcomes for elderly patients undergoing elective orthopaedic surgery [9]. Older patients have been shown to have lower mortality rates and higher rates of discharge to their own home when CGA is used on admission [8]. We also recognize the crucial role of preoperative risk assessment and have subsequently implemented a preoperative assessment service for high-risk elderly patients undergoing major elective urological surgery. This is a monthly outpatient clinic at which a consultant anaesthetist, consultant geriatrician and specialist nurse are in attendance. This aims to improve surgical outcomes through a holistic approach to patient care. The multidisciplinary preoperative assessment service incurs additional costs owing to the extra commitment from a consultant geriatrician and a specialist nurse. Whilst we recognize the necessity of CGA in the preoperative setting, the implementation of this service to high-risk general surgical patients over age 65 is not currently possible within the allocated sessions of a single consultant geriatrician.

Due to limitations in the commissioning of our service our MCOP physician has been restricted to patients over the age of 65. We recognise however that frailty is increasingly being recognised as distinct from age, and may have a greater impact on clinical outcomes. Independent of age frailty is directly correlated with mortality, LOS and readmission within 30 days [10,11,12]. Studies have shown that 16% of younger adults admitted to emergency surgical units were deemed frail, whereas a fifth of older adults undergoing emergency laparotomies were frail [13,14]. It may be that a frailty assessment on admission proves more beneficial at identifying patients in need of additional care over age alone or CGA assessment.

Whilst current standards from the national emergency laparotomy audit (NELA) support early input from MCOP physicians and recommend standardized protocols in postoperative elderly care [15], we are one of the few hospitals with a defined surgical liaison service for elderly patients. An established model of care is lacking in other surgical units across the UK as specific commissioning may be required for the provision of this service. The cost effectiveness will need objective evaluation and this may be one of the rate limiting steps in its implementation. For this reason, proactive engagement in the NELA and its recommendations will be one of the most important drivers towards better care for surgical elderly patients.

We also believe that there needs to be greater emphasis on surgical liaison within the elderly care training program [16]. It will allow trainee geriatricians to gain greater exposure into this subspecialty and deliver care to elderly surgical patients in accordance with these principles even in the absence of the formal liaison service. Equally there should be an increased focus within the surgical training schemes on the impact age an frailty within the peri-operative period [17].

## 5. Limitations

We have presented data from a small single-centre study showing early results from a newly implemented service. As such we have variations in our cohorts and difficulty in providing quantitative data to support our service. To fully assess the efficacy of the new model of care we recognise the need for a large prospective trial across several centres.

## 6. Conclusions

Elderly care input is now a key agenda highlighted by the National Confidential Enquiry into Patient Outcome and Death (NCEPOD), however the evidence to support surgical liaison services remains poorly defined and additional funding for the provision of this service can be limited [3]. Despite a national appetite for the provision of elderly care liaison services only one third of MCOP physicians report providing input into the care of older surgical patients [18]. Whilst we believe that the potential benefits could only be evaluated when a comprehensive preoperative and postoperative service is implemented, our current care model demonstrates that liaison services can be delivered within the existing model without vast expense.

## Figures and Tables

**Table 1:** Peri-operative characteristics and demographics.

		Pre-medicine for the Care of Older People (MCOP) (*n* = 25)	Post-MCOP (*n* = 32)	*p* Value
Age *		69	82	0.001
**Male: Female**		10:15	11:21	0.078
ASA Grade *		2	3	0.005
**Operative severity**	**Major+**	11	17	0.59
	**Major**	3	7	0.48
	**Other**	11	8	0.16

* Median.

**Table 2:** Post-operative outcomes.

	Pre-MCOP *(n* = 25)	Post-MCOP (*n* = 32)	*p* Value
Inpatient MCOP reviews *	0	2	0.0001
**Occupational therapist/Physiotherapist Review**	11	31	0.0001
Length of Stay (days) *	11	16	**0.022**
**Mortality**	1	3	0.623
**Post-Operative ITU Care**	5	9	0.756
**Unplanned Medical Reviews**	16	29	0.488
Delayed Discharge (days) *	3.68	4.75	0.56
**Complications**	6	11	0.56

* Median.

**Table 3:** Qualitative service evaluation.

Qualitative Questionnaire Assessment (*n* = 31)	
Is caring for older people more or less difficult than the younger population? **	4.23
Are elderly care doctors needed to review older people who undergo surgery?^	4.84
Has the introduction of the liaison service made it easier to get medical reviews as compared with before the service was started? ** ** 1: much easier, 5: much more difficult	1.37
^ 1: not needed, 5: very much needed	
